# Evaluation of the Turkey Nutrition and Health Surveys according to the mediterranean adequacy index and sustainability through water footprints

**DOI:** 10.1017/S1368980023001957

**Published:** 2023-12

**Authors:** Nazlıcan Erdoğan Gövez, Şerife Akpınar Şentüre, Şerife Ayten, Eda Köksal

**Affiliations:** Gazi University, Faculty of Health Sciences, Department of Nutrition and Dietetics, Emek, Ankara, 06560, Turkey

**Keywords:** Mediterranean diet, Nutrition, Sustainability, Sustainable nutrition, Water footprint

## Abstract

**Objective:**

Sustainable diet is one of the main factors that support food security, and the Mediterranean diet (MD) one of the sustainable diet models associated with low ecological impact and optimum health results has come to the fore. It was aimed to compare the results of the 2010 and 2017 Turkey Nutrition and Health Studies (TNHS) according to the Mediterranean Adequacy Index (MAI) and in order to evaluate the environmental impact of the current nutritional status in Turkey through water footprints (WF).

**Design:**

The MAI score was calculated using the published results of the 2010 and 2017 TNHS, and the WF have been calculated as indicators of environmental impact.

**Setting:**

Turkey.

**Participants:**

There are no participants.

**Results:**

In the TNHS, there was an increase in the amount of energy provided by foods non-MD in 2017 compared to 2010, with a decrease in the total MAI score. The group with the lowest adherence to the MD in both years was the adult group (MAI_2010_2·74 and MAI_2017_2·31), while the group with the highest adherence was the adolescent group (MAI_2010_3·21 and MAI_2017_2·53). The MAI scores of females were higher than those of males in both years. The males aged 19–64 years had the largest (841 m^3^/year) WF and the females aged 65+ years had the smallest (483 m^3^/year). The food group that contributed the most to WF was meat and meat products (21·0–35·0 %).

**Conclusions:**

Adherence to the MD has decreased due to the increase in the consumption of the Western-type diet in Turkey.

It is estimated that the world’s population will reach 8·5 million by 2030 and 9·7 million by 2050^(1)^. With this increasing global population, ensuring food security has become more important than ever before. Due to the significant effects of food production on the environment, adequate nutrition is not only related to survival and health but also to the environment. Agricultural lands cover 36·9 % of the planet’s land masses^(2)^, and food production is responsible for 30 % of greenhouse gas emissions and 70 % of freshwater use^(3,4)^. Therefore, it is of great importance for countries to adopt sustainable and healthy diet models because of their positive effects on both human health and the environment. Water footprints (WF), carbon footprints and ecological footprints can all be used to determine the environmental effects of diet models^(5)^.

The Mediterranean diet (MD), which is one of these sustainable diet models, is rich in plant-based foods (grains, legumes, oilseeds, fruits and vegetables) and low in red and processed meat. This diet model is also declared by UNESCO to be an intangible cultural heritage with harmonious interactions with agriculture, nutritional practices, the environment and the geography in which Turkey is located^(6)^. In this diet, in which olive oil is adopted as the main source of fat, moderate consumption of fish, seafood, eggs, poultry, dairy products and alcohol is recommended^(7)^. Studies have shown that the MD has a protective effect against CVD, diabetes, metabolic syndrome, cancer and certain neurodegenerative diseases^(8–10)^. Plant-based diet models such as the MD with low quantities of animal-derived nutrients provide numerous benefits for both human and planetary well-being with their beneficial effects on health and positive effects on sustainability^(9)^.

Alberti-Fidanza *et al.* developed the Mediterranean Adequacy Index (MAI) to determine adherence to the MD model based on data from the Seven Countries Study of Cardiovascular Diseases^(11,12)^. The MAI score is calculated by dividing the sum of the energy percentages of typical Mediterranean food groups by the sum of the energy percentages of non-Mediterranean food groups. A higher MAI score indicates better adherence to the MD model^(11)^.

The present research has two aims: the first aim is to compare the results of the 2010 and 2017 Turkey Nutrition and Health Studies (TNHS) according to MAI scores, and the second aim is to evaluate the environmental impact of the current food consumption patterns in Turkey through WF.

## Methods

In this study, the daily intakes of food groups as reported in the 2010 and 2017 TNHS were used to calculate MAI scores, and WF were also evaluated. Both TNHSs, which aimed to determine the nutritional habits and nutritional status in Turkey, are comprehensive surveys conducted in the country and constitute an important data source. The survey is performed periodically to assess population nutritional and health status. In the 2010 TNHS, a total of 17 452 people aged 15 years and over were evaluated, while 24 906 people participated in the 2017 TNHS. In both years, the TNHS sample sizes were determined in such a way that the results of the study could be evaluated reliably according to gender and age groups and would be representative throughout the country. Data on food consumption were obtained using the 24-h dietary recall method^(13,14)^.

The MAI score is evaluated with four basic parts. These are the carbohydrate food group (bread, cereals, legumes and potatoes), protective food group (vegetables, fruits, fresh legumes, fish, alcoholic beverages, especially wine, and vegetable oils), animal food group (milk, cheese, meat, eggs, animal fat, margarine and butter) and sweet food group (sugary drinks, cakes, pastries, cookies and sugar). The MAI score is calculated by dividing the energy provided by the carbohydrate and protective food groups by the energy provided by the animal and sweet food groups^(12)^. In this study, some adjustments were made in the calculation of MAI scores, taking into account the data provided by the 2010 and 2017 TNHS. Legumes and oilseeds were evaluated together in the 2017 TNHS to facilitate appropriate comparisons because legumes had been given together with oilseeds in the 2010 TNHS^(13,14)^. It was thought that this would not pose any problems since the food groups related to the MD model were included in the MAI evaluations. Since alcohol consumption is limited in Turkey society and excessive alcohol consumption can have negative effects on health, alcohol was not included in the MAI calculations^(13,14)^. In addition, fish consumption could not be evaluated separately in the 2010 TNHS, since fish consumption was very low in the considered population. As a result, the carbohydrate food group included bread, cereals and legumes (including oilseeds); the protective food group included vegetables (including fresh legumes), fruits (fresh and dried) and vegetable oils; the animal food group included dairy products, meat and meat products, eggs, and fats; and the sweet food group included sugar and sugary foods. The formula for the calculation is presented below:

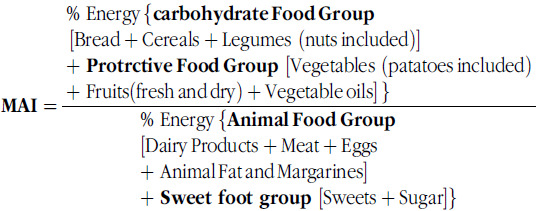




The total energy contents of the foods in these four groups according to age groups and gender were calculated with the Nutrition Information System (BeBiS) 9.0 program ^(15)^. There is no cutoff point for MAI scores, and evaluations were only performed for observed changes.

The Extended Water Footprint Calculator developed by Hoekstra *et al*. was also used in this study to calculate WF as sustainability and environmental impact indicators^(16)^. However, since WF components such as vegetable and fruit consumption, potato consumption, and coffee and tea consumption are not given separately in the 2010 TNHS results, calculations were only made for the 2017 TNHS results.

## Results

When the 2010 and 2017 data from the TNHS were analysed based on MAI food groups according to age groups and gender, it was observed that the consumption of bread in the carbohydrate food group decreased during that period among female participants over 65 years of age and the general group of participants over 65 years of age. The consumption of cereals increased in only the age group of 19–64 years and decreased for the other age groups. Legume consumption increased among all age groups and both genders. When the protective food group was examined, vegetable consumption was seen to have increased in the age group of 15–18 years, while fruit consumption increased among those aged 65 years and over and decreased among the other age groups. The consumption of vegetable oil increased in the adult age group and among male participants aged 15–18 years, while it decreased among other age groups and female participants. Considering the consumption of animal foods in the non-MD group, it was determined that the consumption of milk decreased among male participants aged 65 years and older, while egg consumption decreased among male participants aged 15–18 years; increases were observed for all other age groups and female participants. The intake of sweet foods decreased among males aged 15–18 years and in total, as well as in the age group of 65 years and over, while it increased in the other groups (Table 1).

**Table 1:** Mean intakes of MAI food groups according to 2010 and 2017 TNHS

Food groups	15–18 years			19–64 years			65+ years		
	M	F	T	M	F	T	M	F	T
Carbohydrate food group									
Bread (g/d)									
2010	266·4	157·9	209·6	241·2	153·9	188·2	192·1	150·7	166·2
2017	290·1	175·2	230·8	247·6	151·5	194·7	198·1	136·2	162·6
Cereals (g/d)									
2010	95·9	80·1	87·6	72·4	63·9	67·2	55·3	45·7	49·3
2017	93·3	74·2	83·4	84·4	67·9	75·3	40·9	44·5	43·0
Legumes (raw, dried) (g/d)									
2010	15·9	13·4	14·6	16·6	13·3	14·6	11·9	10·9	11·3
2017	24·7	23·1	23·9	30·9	24·9	27·6	24·2	17·8	20·5
Protective food group									
Vegetables (g/d)									
2010	155·4	159·7	157·7	343·4	346·7	345·4	359·1	313·3	330·4
2017	243·0	210·2	226·1	259·6	260·0	259·8	252·8	250·5	251·5
Fruits (fresh and dried) (g/d)									
2010	279·8	287·5	283·8	190·8	192·6	191·9	199·5	174·0	183·5
2017	136·5	136·8	136·6	160·7	152·6	156·2	203·2	182·3	191·2
Vegetable oils (g/d)									
2010	20·5	21·5	21·0	22·1	20·3	21·0	19·9	17·3	18·3
2017	22·0	19·3	20·6	23·7	21·1	22·3	19·2	17·0	17·9
Animal food group									
Dairy products (g/d)									
2010	176·2	130·8	152·4	163·5	143·4	151·3	186·6	141·0	158·0
2017	225·3	187·4	205·8	206·1	169·7	186·1	184·7	168·6	175·5
Meat (g/d)									
2010	62·1	36·1	48·5	84·4	46·6	61·5	43·2	31·8	36·1
2017	99·9	65·6	82·2	116·7	63·5	87·4	70·3	48·6	57·9
Eggs (g/d)									
2010	28·9	19·8	24·1	26·2	20·9	23·0	20·3	13·9	16·3
2017	24·3	20·3	27·1	36·0	29·6	32·5	29·2	23·4	25·9
Animal fats and margarine (g/d)									
2010	12·7	7·9	10·2	10·4	7·8	8·8	6·5	4·7	5·4
2017	15·4	9·8	12·5	14·1	9·6	11·6	9·5	6·7	7·9
Sugar and sweet food group (g/d)									
2010	31·8	26·3	28·9	35·0	27·6	30·5	29·3	20·6	23·8
2017	29·8	35·0	27·3	36·2	27·5	31·4	27·9	20·2	23·5

M, male; F, female; T, total.

The amount of energy provided by carbohydrate and protective food groups, which are classified as MD foods according to the MAI, decreased in the entire age group of 65 years and over, in the age group of female participants aged 15–18 years, and in the general age group of 15–18 years. On the other hand, the energy obtained from the animal and sweet food groups including non-MD foods increased among all age groups and both genders. In addition to the analysis performed for the age groups, the MAI score components were also evaluated by general means. According to these results, energy intake from foods included in the MD model increased in most age groups and both genders. Moreover, energy intake from foods not included in the MD was also increased. Thus, total energy intake increased between 2010 and 2017 (Table 2).

**Table 2:** Energy contents of MAI components in 2010 and 2017 TNHS

	15–18 years			19–64 years			65+ years			15+ years		
	M	F	T	M	F	T	M	F	T	M	F	T
Group 1 (kcal/d)												
2010	1406·3	1094·7	1242·8	1257·1	992·0	1096·0	1053·5	870·0	939·0	1239·3	978·0	1081·5
2017	1420·1	1040·4	1224·0	1335·6	1000·0	1151·1	1029·1	841·2	903·6	1288·8	973·1	1111·3
Group 2 (kcal/d)												
2010	458·2	322·3	387·0	476·6	351·4	400·5	367·3	266·4	304·1	458·0	335·0	383·6
2017	548·4	449·0	483·0	595·8	417·2	497·3	436·3	334·2	378·0	567·4	403·5	476·1
Total energy intake (kcal/d)												
2010	1864·5	1417·0	1630·0	1734·0	1343·4	1496·5	1420·7	1136·4	1243·1	1697·3	1313·0	1465·1
2017	1968·4	1489·4	1707·0	1931·4	1417·3	1648·4	1465·4	1175·4	1281·6	1856·2	1376·6	1587·4

M, male; F, female; T, total.

Group 1: Energy from carbohydrate food group + protective food group.

Group 2: Energy from animal food group + sweet food group.

Total energy intake: Group 1 + Group 2.

**Fig. 1:**
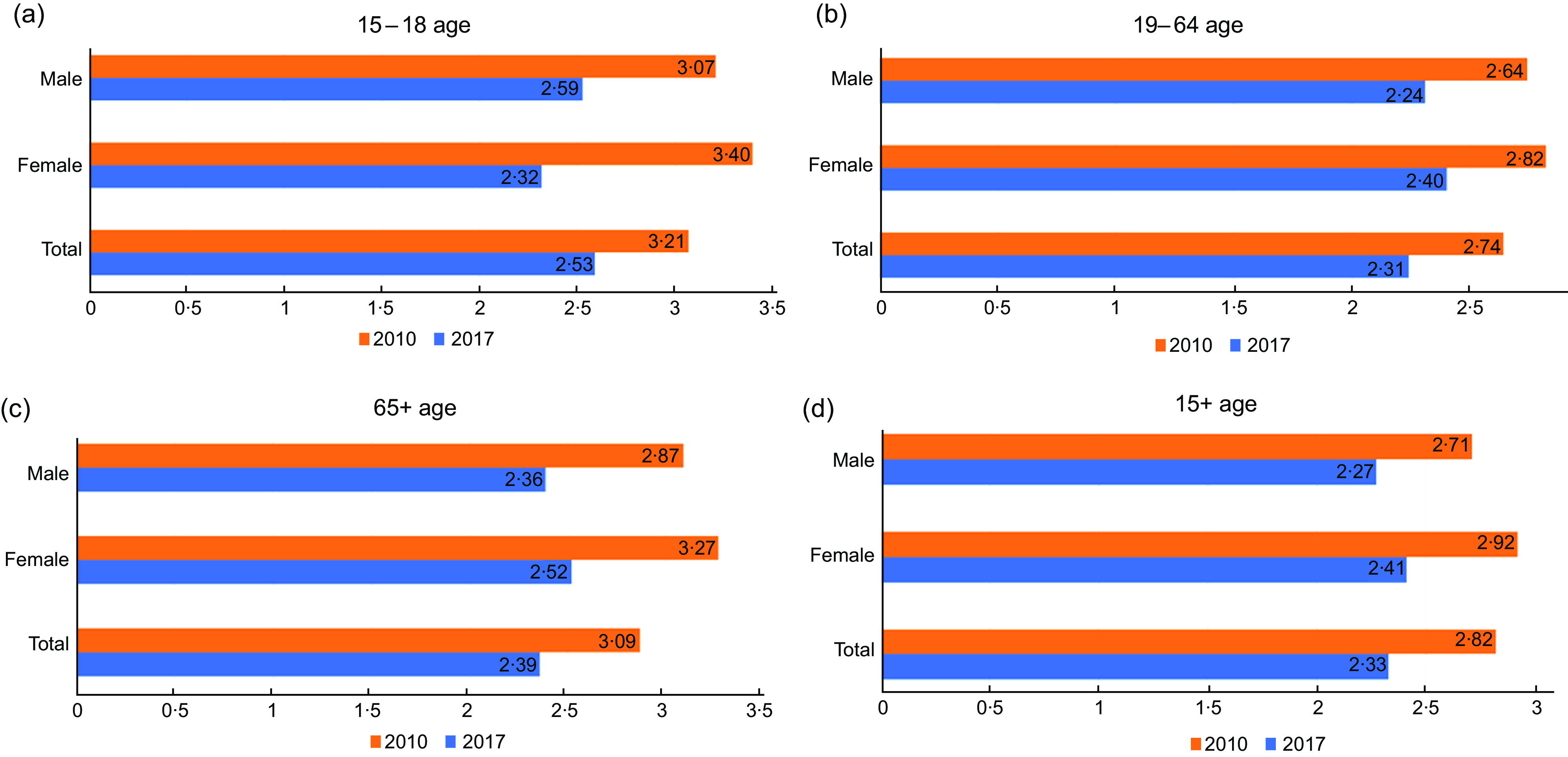
MAI score comparisons. Figure 1 shows the MAI scores for the 2010 and 2017 TNHS by age and gender. MAI scores were calculated based on means, taking into account age groups and the sizes of the populations in those age groups. (a) MAI scores calculated for the 2010 and 2017 TNHS findings for 15–18 age group. (b) MAI scores calculated for the 2010 and 2017 TNHS findings for 19–64 age group. (c) MAI scores calculated for the 2010 and 2017 TNHS findings for 65+ age group. (d) MAI scores calculated for the 2010 and 2017 TNHS findings for 15+ age group

Figure 1 shows the MAI scores calculated for the 2010 and 2017 TNHS findings by age and gender. When the MAI scores of the 2010 TNHS and 2017 TNHS were compared, it was determined that the scores decreased among all age groups and both genders. In addition, when the 2010 and 2017 TNHS results were compared according to age groups, the highest scores in both 2010 and 2017 were obtained for the age group of 15–18 years (3·21 and 2·74, respectively), while the lowest scores were obtained for the age group of 19–64 years (3·21 and 2·74, respectively) (Fig. 1). Finally, the general MAI scores for the TNHS were calculated, and it was concluded that MAI scores decreased over the years for both genders and the total population (for 2010 and 2017, MAI_Male_: 2·71 and 2·27; MAI_Female_: 2·92 and 2·41, respectively; MAI_Total_: 2·82 and 2·33).

**Fig. 2:**
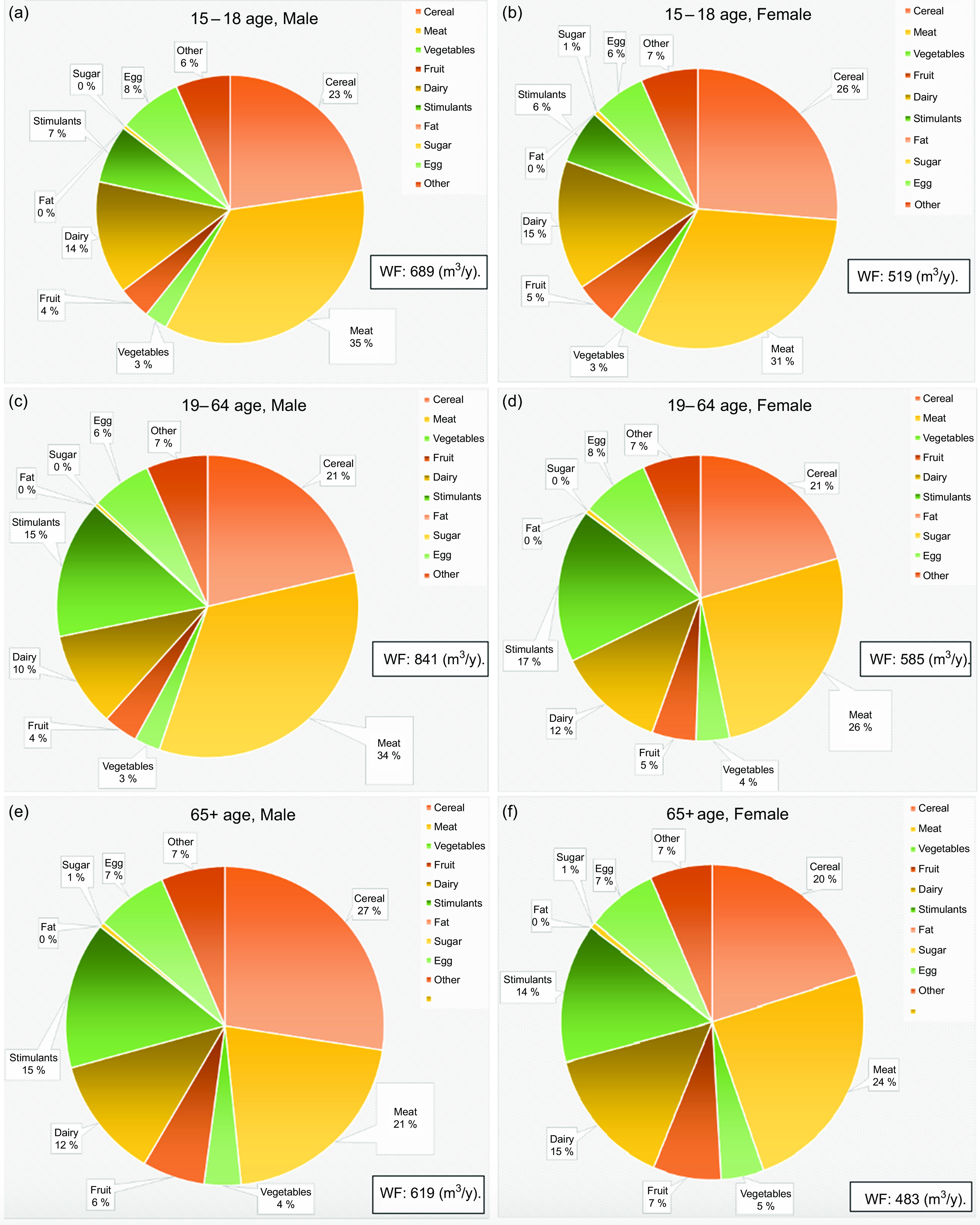
Evaluation of individuals’ water footprints (WF) according to 2017 TNHS food consumption results. (a) Total water footprint and contribution of food groups to water footprints for 15–18 years of age, male. (b) Total water footprint and contribution of food groups to water footprints for 15–18 years of age group, female. (c) Total water footprint and contribution of food groups to water footprints for 19–64 years of age group, male. (d) Total water footprint and contribution of food groups to water footprints for 19–64 years of age group, female. (e) Total water footprint and contribution of food groups to water footprints for 65+ years of age group, male. (f) Total water footprint and contribution of food groups to water footprints for 65+ years of age group, female

To evaluate the environmental impact of current food consumption patterns in Turkey, WF were calculated for the 2017 TNHS results by age group and gender as illustrated in Fig. 2. When these results were examined, it was seen that male participants aged 19–64 years had the largest WF (841 m^3^/year), while women aged 65 years and over had the smallest (483 m^3^/year). The food group that contributed the most to the WF values of all groups was the meat and meat products group (21·0–35·0 %). After meat and meat products (21·0–35·0 %), cereals (21·0–27·0 %), and milk and other dairy products (10·0–15·0 %) had the largest contributions to the WF values. The consumption of tea and coffee, defined here as stimulants, affected the WF of male and female participants aged 15–18 years by 6·0–7·0 % and those of male and female participants aged 19–64 years and 65 years and over by 15·0–17·0 %. When the contribution of egg consumption to WF values was examined, it was found to affect all groups similarly at rates of 6·0–8·0 %. Levels of fruit and vegetable consumption affected the WF values at very low rates (fruits: 5–7 %; vegetables: 3–5 %), while sugar affected WF values the least (0–1·0 %).

## Discussion

This study considered the effects on planet and human health and compliance with the MD evaluated using the MAI score and WF and using population-based survey data on nutrition and health. In light of data obtained from the 2010 and 2017 TNHS, the amount of energy provided by MD food groups decreased between those years in Turkey in the entire elderly group (65 years and over), female participants aged 15–18 years, and the entire age group of 15–18 years. The energy obtained from the animal food and sweet food groups, which are incompatible with the MD, increased in all age groups and for both genders. When the MAI scores for the TNHS results from 2010 and 2017 were compared, it was determined that average scores had decreased for all age groups and both genders. While there was a decrease in the amount of energy provided by Mediterranean-type food groups, the amount of energy supplied by non-Mediterranean foods increased, and this resulted in a decrease in MAI scores. This change is thought to be related to the shift of nutritional systems in developing countries from traditional diet models to Western diet patterns, which has been reported in recent years^(17,18)^.

In a previous study, changes in MAI scores as determined by the FAO Food Balance Sheets of forty-one countries in total, including Turkey, were evaluated between 1960 and 2011. It was found that the MAI scores of six Mediterranean countries and ten non-Mediterranean countries increased in the periods between 2000–2003 and 2004–2011; on the contrary, in the rest of the countries, including Turkey, decreases in MAI scores were seen. In the same study, Turkey’s MAI scores in the periods of 1961–1965, 2000–2003, and 2004–2011 were reported as 5·03, 2·80, and 2·37, respectively^(19)^. In our study, the average MAI score similarly decreased between 2010 and 2017, with scores of 2·79 and 2·33, respectively.

In our study, when MAI scores were compared according to the 2010 and 2017 TNHS results, the highest score in 2010 was obtained for the age group of 15–18 years (3·21), while the lowest score was obtained for the age group of 19–64 years (2·74). For the 2017 TNHS, the highest score was again obtained for the age group of 15–18 years (3·21) and the lowest score was obtained for the age group of 19–64 years (2·74). Thus, the group with the lowest adherence to the MD in both years was the adult group (19–64 years) and the group with the highest adherence was adolescents (15–18 years). Similarly, in the study conducted by Sureda *et al*. (2018), the compliance of participants aged 12–17 years to the MD was found to be higher than that seen among individuals aged 18–65 years^(20)^. Adolescents tend to underreport sugar and fast-food consumption, which may make their adherence to the MD seem higher than it is, and it should be considered that this factor may have affected the results of our study, as well. However, another study in the literature presented opposing findings; the adherence of individuals aged 60 years and over to the MD was reported to be higher than that of the age groups of 46–60 years and 18–30 years, while it was lower than that of the age group of 31–45 years. As age increased within these groups, adherence to the MD was found to increase significantly (*P* < 0·05), with the increase being especially evident at the age of 45 years and above^(21)^.

When our results are evaluated according to gender, the MAI scores of female participants are found to be higher than those of male participants for all age groups in 2010, and for 2017, the MAI scores of female participants are higher than those of male participants in all age groups except 15–18 years. Thus, according to the findings of our study, women’s adherence to the MD is generally higher than that of men. Similarly, in another study, it was found that women’s adherence to the MD was higher than that of men in the adult age group (*P* < 0·05)^(21)^. However, other studies in the literature have presented opposing results^(20,22–24)^. The level of adherence to the MD may differ between men and women according to age^(25)^. One study found that differences in adherence to the MD between male and female participants decreased with age^(26)^. On the other hand, it was stated that there was no relationship between gender and adherence to the MD in a systematic review^(27)^. In light of these results, it can be assumed that nutrition may be affected by many different ecological and demographic factors; therefore, changes in MAI scores may be due to differences in the population base in which a given study is conducted.

In recent years, diets have been evaluated in terms of sustainability as well as human health. Ensuring a healthy and sustainable diet for the world’s growing population poses a major challenge^(28)^. It has been argued that food policy should shift from the traditional approach focusing on nutrition guidelines and food safety measures, nutrients, and health to an approach that is sustainable and takes into account the environmental, economic, and social dimensions of diets^(29)^. The MD was presented as a part of this solution^(30)^. The amount of surface and groundwater used in production processes is evaluated as blue water, the amount of water corresponding to rainwater used in production processes is evaluated as green water, and the amount of water required to clean the dirty water arising from production or the supply chain is evaluated as grey water. In this context, nutrient intake (source/groundwater required for raising crops) creates a green WF with the rainwater that can be used for crops^(31)^. In a previous study, the green + blue WF value determined according to FAOSTAT data in Turkey was found to be 3812 liters per person per d. Compared to the WF of the Mediterranean and EAT-Lancet (Planetary Health Diet) diets, which are accepted as sustainable diet models, the WF of Turkey’s current diet model is 26 % higher than that of the MD (2819 liters per person per d) and 38 % higher than that of the EAT-Lancet diet (Planetary Health Diet) (2353 liters per person per d)^(32)^.

According to Turkey’s Water Footprint Report (2014), while the per capita WF was 1642 m^3^/year between 1996 and 2005, it increased to 1977 m^3^/year between 2006 and 2011. It has been determined that the majority of the WF of production in Turkey originates from agriculture, with cereals (38 %) contributing the most and vegetables and legumes (2 %) contributing the least to the WF in the agricultural sector^(33)^.

Similarly, in a study conducted in China, it was determined that cereals contributed the most to the WF due to high rice consumption, followed by meat and meat products, and then vegetables^(34)^. Although cereal consumption is high in Turkey, the consumption amounts are lower than those of China^(14,34,35)^. In our study, when the contribution of food groups to the WF was evaluated, it was determined that meat and meat products were the highest contributors, followed by cereals. According to FAOSTAT data, the groups that contribute the most to the WF in Turkey are cereals and potatoes, meat and meat products, and milk and dairy products, respectively^(32)^. While the FAOSTAT data are indirect data obtained by dividing data for the total population by registered production and export and import items, the TNHS data were obtained by evaluating real individual consumption while also taking into account the amount of waste generated at home^(36)^. This is the likely source of the difference between the data reported by FAOSTAT and the findings presented in our study. In a study conducted with adult participants, meat and meat products (33·06 %), milk and milk products (21·30 %), cereals (13·44 %), fruits (7·16 %), and vegetables (3·90 %) contributed the most to WF^(37)^. In addition to these findings, it has been reported in many studies that the contributions of foods of animal origin to both greenhouse gas emissions and WF are higher than those of other food groups^(4,38)^. A systematic review and meta-analysis of dietary patterns and WF concluded that with a decrease in the consumption of those foods, WF will decrease significantly; dietary models that completely exclude red meat and meat products have been shown to create approximately 25 % decreases in total WF and approximately 12 % decreases in blue WF^(39)^. Although the contribution of meat and meat products to the WF was found to be highest among the considered food groups in our study, the consumption levels of meat and meat products in Turkey are below the recommended amounts. Thus, it seems that the WF contributions of meat and meat products are higher than those of other foods regardless of the amount of meat consumption. In addition, according to our findings, since the MD is a sustainable diet model, it can be said that low MAI scores reflect increased ecological effects of dietary patterns.

There are some limitations of the present study and its findings. First of all, the 2010 and 2017 TNHS entail some inconsistencies between the collected data and the evaluation of findings. As the present study was not based on raw data, we were limited by an inability to perform some statistical analyses. If we had raw data, we could better separate both WF and dietary data. In addition, fish consumption could not be evaluated separately in the 2010 TNHS and since fish and alcohol consumption was very low in the Turkish population, they are not included in the MAI calculations. Since the consumption amounts are low, we think that it did not significantly affect the results. On the other hand, the strongest aspect of this study is that it is the first study to calculate MAI scores and WF values from TNHS data. Although a previous study from Turkey reported MAI scores, it was an indirect study that evaluated data obtained from food balance sheets, while our study offers more direct findings. However, it must be emphasised that to determine the changes over the years, the elaboration of data or even direct evaluations of raw data will give the most optimal results.

### Conclusion

The MD model has attracted much interest with its beneficial effects on health as well as its positive effects on sustainability and low ecological impact with low levels of foods of animal origin and higher plant-based contents. In this study, when MAI scores and WF values were evaluated together, it was seen that the results were consistent and supported each other. It is expected that environmental impacts will be reduced by replacing existing dietary patterns with more sustainable diet models, such as the MD. However, the public may be reluctant to embrace this message due to a lack of information about the environmental impacts of current dietary patterns. In this context, it is important to organise training sessions that aim to inform people about the environmental effects of diets in addition to their health effects to ensure that a livable world is left for future generations.
